# Significance of nerve growth factor overexpression and its autocrine loop in oesophageal squamous cell carcinoma

**DOI:** 10.1038/sj.bjc.6603255

**Published:** 2006-07-11

**Authors:** S Tsunoda, T Okumura, T Ito, Y Mori, T Soma, G Watanabe, J Kaganoi, A Itami, Y Sakai, Y Shimada

**Affiliations:** 1 Department of Surgery and Surgical Basic Science, Graduate School of Medicine, Kyoto University, Kawaracho 54 Shogoin Sakyo-ku, Kyoto 606-8507, Japan

**Keywords:** NGF, TrkA, p75NTR, oesophageal cancer, immunohistochemistry, autocrine

## Abstract

Nerve growth factor (NGF) is overexpressed not only in nervous system, but also in several types of cancers. However, the role of NGF in oesophageal squamous cell carcinoma (OESCC) remains unclear. Here, we show the first evidence of NGF-TrkA autocrine loop and clinical significance of NGF overexpression in OESCC. Immunohistochemical study of 109 OESCC specimens revealed that NGF overexpression, found in 63 out of 109 patients (57.8%), was associated with lymph node metastasis, distant metastasis, higher TNM stage, poorer tumour differentiation, and poorer survival. NGF overexpression was also associated with strong expression of TrkA and negative expression of low-affinity neurotrophin receptor (p75NTR). Semiquantitative reverse transcription–polymerase chain reaction (RT–PCR) of 19 surgical specimens showed upregulation of NGF mRNA in 17 out of 19 (89%) patients. All five OESCC cell lines tested *in vitro* secreted detectable NGF in enzyme-linked immunosorbent assay, and expressed TrkA and p75NTR on RT–PCR and Western blot. The motility of HSA/c, one of the OESCC cell lines overexpressing NGF, was significantly decreased by either neutralising anti-NGF antibody, an inhibitor of TrkA, or NGF-small interfering RNA in transwell migration assay. Our findings suggest that NGF is of potential interest not only as a prognostic factor, but also as a novel therapeutic target in OESCC.

Oesophageal squamous cell carcinoma (OESCC) is one of the most lethal malignancies in the world including Japan, with a 5-year survival rate of 20–30% after curative surgery (Isono *et al*, 1991; Pisani *et al*, 1999). One of the reasons for poor prognosis is that OESCC is frequently associated with extensive local invasion or regional lymph node metastasis, even at initial diagnosis. Improved treatments derived from a better understanding of the biological basis of OESCC are now awaited.

We previously reported that the low-affinity neurotrophin receptor (p75NTR) was expressed in a progenitor cell fraction of human oesophageal keratinocyte (Okumura *et al*, 2003). Nerve growth factor (NGF), one of the ligands of p75NTR, was originally isolated for its ability to stimulate both the survival and differentiation of peripheral neurons, later becoming the archetypal member of the neurotrophin family of polypeptides (Levi-Montalcini, 1987; Barbacid, 1995). A major biological function of NGF is the maintenance and survival of postmitotic neurons, suggesting it may be useful for the treatment of neurodegenerative diseases. In addition to its role in the development and maintenance of neuronal cells, recent studies have shown that NGF and its receptors are found and sometimes even overexpressed outside the nervous system, where it may promote cancer cell proliferation, growth, and invasion in several types of cancer such as breast, pancreas, and prostate cancer (Pflug *et al*, 1995; Walch and Marchetti, 1999; Sakamoto *et al*, 2001a, 2001b; Schneider *et al*, 2001; Zhu *et al*, 2001, 2002; Kishibe *et al*, 2002; Davidson *et al*, 2003; Dolle *et al*, 2003).

Nerve growth factor generates intracellular signals by interacting with two classes of membrane receptors: the TrkA proto-oncogene product p140trkA, which possesses intrinsic tyrosine kinase activity, and a secondary receptor, p75NTR, which is a member of the tumour necrosis factor-receptor family (Sofroniew *et al*, 2001). Nerve growth factor has also been found to be an autocrine survival factor for B lymphocytes (Torcia *et al*, 1996), and Dolle *et al* (2003) reported that NGF is involved in an autocrine loop in breast cancer. In oesophageal cancer, however, only one small clinical study on NGF expression has been reported (Zhu *et al*, 2000), which is very different from other studies in that they showed downregulation of NGF in tumours with poorer differentiation and in advanced stage. Thus, the significance of NGF expression in OESCC remains unclear.

In this study, to determine whether NGF plays a role in OESCC, we examined the expression of NGF, TrkA and p75NTR in 109 surgical specimens of OESCC. We also evaluated the role of NGF in OESCC cell lines.

## MATERIALS AND METHODS

### Patients and surgical specimens

Frozen tissue specimens and paraffin-embedded sections were obtained from surgically resected specimens from patients with primary OESCC. All patients underwent surgery at the Kyoto University Hospital from 1984 to 2001. Among the 109 patients, 18 were women and 91 were men. The median age of the patients was 63 years (range, 39–84 years). The median follow-up time of survival was 40 months (range, 2–201 months). Information on gender, age, stage of disease, and histopathological factors were abstracted from the patients' medical records. All tumours were confirmed to be OESCC by pathologists in the Department of Pathology at the Kyoto University Hospital. All of the cases were staged according to the *TNM Classification of Malignant Tumours*, Sixth edition, issued in 2002 (Sobin *et al*, 2002). Of the 109 patients, 15 patients (13.4%) were in stage I, 33 patients (30.3%) in stage IIA-B, 39 patients (35.8%) in stage III, and 22 patients (20.2%) were in stage IV. In this series, all cases with a positive M factor had distant lymph node metastasis and there was no organ metastasis. Written informed consent has been obtained from all the patients for surgery and for the use of the resected samples for research. The study protocol has been approved by the Institutional Review Board of Kyoto University.

### Immunohistochemical staining

Paraffin-embedded 4-*μ*m-thick serial sections were autoclaved at 121°C in Target Retrieval Solution (Dako Cytomation, Kyoto, Japan) for 5 min, subjected to paraffin removal, and rehydrated. The sections were immunostained with the Envision Plus kits/HRP/DAB (Dako Cytomation) as recommended by the supplier. Primary antibodies were diluted to 1 : 50 for anti-NGF rabbit polyclonal antibody (sc-548, Santa Cruz Biotechnology Inc., CA, USA), 1 : 400 for anti-TrkA rabbit polyclonal antibody (sc-118, Santa Cruz Biotechnology Inc.), and 1 : 50 for anti-human p75NTR monoclonal antibody (clone NGFR5; Dako Cytomation) with 1% bovine serum albumin (BSA) (Nacalai Tesque Inc., Kyoto, Japan) in phosphate-buffered saline (PBS), and the sections were incubated for 1 h at room temperature (NGF) or overnight at 4°C (TrkA and p75NTR). After rinsing, the sections were incubated with secondary antibody, counterstained with Mayer's hematoxylin, dehydrated, and mounted. Nerve growth factor immunoreactivity in lymphocytes was used as an internal positive control in every section. Sections incubated with blocking peptide (sc-548p, Santa Cruz Biotechnology Inc.) served as a negative control for the evaluation of NGF immunohistochemical staining.

### Evaluation of immunohistochemical staining

Nerve growth factor was expressed diffusely in all OESCC, and NGF staining intensity was evaluated in five areas of each slides and graded into two groups; strong (stronger cytoplasmic staining than smooth muscle of oesophagus) and weak expression (weaker than smooth muscle cells). TrkA was expressed in the cytoplasm and the membrane of OESCC. TrkA staining intensity was graded into two groups; strong (positive membrane staining in more than 30% of OESCC or/and intense staining in cytoplasm of OESCC) and weak/negative expression. p75NTR staining intensity was graded into two groups; positive (positive membrane staining in more than 10% of OESCC) and negative (staining less than 10%). All slides were evaluated independently by two investigators (ST and TO or YM) without any prior knowledge of each patient's clinical information. If the opinions of the two investigators differed, agreement was reached by careful discussion.

### Statistical analysis

The statistical significance of differences in NGF expression levels and clinicopathologic factors or immunohistochemical staining results of TrkA and p75NTR were analysed with *χ*^2^ tests. Overall survival was defined as the duration of survival from the date of the operation to the date of death because of cancer. The Kaplan–Meier method was used to determine the probability of survival, and data were analysed with the log-rank test. The Tukey–Kramer multiple comparison test was used to evaluate the results of migration assays. The software package StatView for Windows version 5 (SAS Institute, Cary, NC, USA) was used for all analyses. *P*-values <0.05 was considered statistically significant.

### Extraction of total cellular RNA and semiquantitative reverse transcription–polymerase chain reaction

Total RNA was extracted from frozen stored tissues of OESCC or from cultured cells using acid guanidinium thiocyanate–phenol–chloroform method. The amount of RNA extracted from each sample was spectrophotometrically measured using a Gene Quanto pro (Amersham Biosciences, Piscataway, NJ, USA). Then, precisely 2.5 *μ*g from each of the extracted RNA samples was used for first-strand cDNA synthesis using a First Strand cDNA Synthesis kit (Amersham, Buckinghamshire, UK) according to the manufacturer's instructions. Specifically, TE solution was added to 2.5 *μ*g of extracted RNA to adjust each of the reaction mixture to the final volume of 8 *μ*l and the mixture was denatured in 65°C for 10 min and incubated on ice for 2 min. Denatured samples were mixed with 5 *μ*l of Bulk First-Strand Reaction Mixes, 1 *μ*l of DTT solution, and 1 *μ*l of pd(N)_6_ primer and incubated in 37°C for 60 min and 95°C for 5 min for cDNA synthesis. Next 1 *μ*l of the first-strand cDNA, 1 *μ*l of sense template (10 *μ*M), 1 *μ*l of antisense template (10 *μ*M), 12.3 *μ*l of double distilled water, 0.6 *μ*l of 50 mM MgCl_2_, 2 *μ*l of 10 × polymerase chain reaction (PCR) Rxn buffer (Invitrogen Corporation, Carlsbad, CA, USA), 2 *μ*l of 2 mM dNTP mix, and 0.1 *μ*l of *Taq* DNA polymerase (Invitrogen Corporation) were used in a total volume of 20 *μ*l to perform semiquantitative reverse transcription–polymerase chain reaction (RT–PCR). Then, PCR cycles were adjusted to make intensity of *β*-actin band from respective samples to be nearly equal. Because the band intensity of NGF turned out to be much lower than that of *β*-actin, on the average, about 30 cycles needed to be run to clarify the difference in expression levels of NGF and TrkA in different samples by RT–PCR. For p75NTR, 25 cycles of PCR were performed for semiquantification. PCR protocol was as follows: 1 min denaturation at 94°C, 1 min annealing at 62°C (NGF) or 66°C (TrkA) or 54°C (p75NTR), and 1 min elongation at 72°C. Amplification products were separated on 2% agarose gels and visualised by ethidium bromide staining. A single 69-bp band amplified with *β*-actin primers from respective cDNA served as an internal control. Then, the signal intensity of each sample was calculated with the ImageJ program version 1.36 (NIH, USA), and the ratio of NGF (or TrkA, p75NTR) to *β*-actin was calculated. Next, NGF (or TrkA, p75NTR) expression in each specimen was evaluated by computing the ratio of NGF (or TrkA, p75NTR) band intensity in tumour to that in the corresponding normal epithelium (T/N ratio). The following PCR primers were used: NGF (403 bp) forward primer was 5′-CCACACTGAGGTGCATAGCGTAA-3′ and reverse primer was 5′-AGATGGGATGGGATGATGACCGCT-3′; TrkA (219 bp) forward primer was 5′-TCCGCCTCCATCATGGCTGCCTT-3′ and reverse primer was 5′-CCCAAACTTGTTTCTCCGTCCACA-3′; p75NTR (230 bp) forward primer was 5′-TGAGTGCTGCAAAGCCTGCAA-3′ and reverse primer was 5′-TCTCATCCTGGTAGTAGCCGT-3′; and *β*-actin (69 bp) forward primer was 5′- CCTGGCACCCAGCACAAT-3′ and reverse primer was 5′-GCCGATCCACACGGAGTACT-3′.

### Cell cultures

Five human OESCC cell lines (KYSE-150, KYSE-170, KYSE-1170, HSA/c, and SUm/c) were used. These cell lines have been established in our department and cultured in Ham's F-12/RPMI 1640 with 2% (KYSE series) or 5% (HSA/c and SUm/c) fetal bovine serum (FBS) as described previously (Shimada *et al*, 1992; Nakaji *et al*, 1999). For positive control, human breast cancer cell line, MCF-7 was obtained from the Japanese Collection of Research Bioresources and cultured in Eagle's minimum essential medium with nonessential amino acid with 1 mM pyruvate, 1.5 g 1^−1^ NaHCO_3_, 0.01 g l^−1^ insulin, and 10% FBS. Cells were incubated at 37°C in a humidified atmosphere of 5% CO_2_ in air and subconfluent cells were used in all experiments.

### Western blot analysis

Cells were lysed in a sample buffer (10% glycerol, 20 mM Tris-HCl, pH 7.5, 1% NP-40, 100 mM NaF, 1 mM Na3VO4, 1 mM phenylmethyl sulfonyl fluoride; Complete Mini: Roche Diagnostics GmbH, Mannheim, Germany) on ice. After sonication and centrifugation, the supernatant was used for assay. The protein concentration was estimated by the Bradford method using BCA Protein Assay Reagent (Pierce, Rockford, IL, USA). After boiling, cell lysate (80 *μ*g) was electrophoresed on 15–25% gradient polyacrylamide gel (Daiichi Pure Chemicals, Tokyo, Japan) and transferred to polyvinylidine diflouride membranes (Immobilon, Millipore, Bedford, MA, USA) for NGF. For TrkA and p75NTR, 25 *μ*g of cell lysate was electrophoresed on 2–15% gradient polyacrylamide gel (Daiichi Pure Chemicals). Membranes were blocked with 4% BSA in 0.1% Tween-20 in Tris-buffered saline (20 mM Tris, 150 mM NaCl, pH 7.6) for 1 h in room temperature. A rabbit anti-NGF polyclonal antibody (sc-548, Santa Cruz Biotechnology Inc.) was used as primary antibody against NGF. The membrane was incubated with primary antibody (diluted 1 : 800) at 4°C overnight. The membrane was subsequently incubated at room temperature for 1 h with horseradish peroxidase-linked goat anti-rabbit IgG (Zymed, San Francisco, CA, USA) (diluted 1 : 4000). The final detection of specific proteins was carried out with the use of enhanced-chemiluminescence (ECL-plus) reagents (Amersham). For p75NTR and TrkA, a rabbit anti-human p75NTR polyclonal antibody (G332A, Promega, Madison, WI, USA) (diluted 1 : 1000) and a rabbit anti-TrkA polyclonal antibody (sc-118, Santa Cruz Biotechnology Inc.) (diluted 1 : 200) were used, respectively. For phosphorylated TrkA, a mouse monoclonal anti-p-TrkA antibody (sc-8058, Santa Cruz Biotechnology Inc.) (diluted 1 : 200) was used. Lysate from the breast cancer cell line MCF-7 was used as a positive control for NGF (Dolle *et al*, 2003), TrkA, and p75NTR (Descamps *et al*, 2001b).

### Enzyme-linked immunosorbent assay

Cells were seeded into 50-mm dishes in Ham's F-12/RPMI 1640 with 2% (KYSE series) or 5% (HSA/c, SUm/c) FBS, until 70% confluent. Then, the cells were rinsed with PBS and the medium was replaced with serum-free Ham's F-12/RPMI 1640 and maintained for another 48 h. The medium was clarified by centrifugation at 3000 r.p.m. for 15 min and kept at −80°C for further experiments. In the meantime, cell counts were determined, followed by extraction of cellular RNA. To detect NGF in the medium, the Emax ImmunoAssay System (Promega) was used according to the manufacturer's instructions. All determinations were in triplicate, and data are the means of three independent experiments.

### Immunofluorescence staining and confocal microscopy

HSA/c cells were cultured onto collagen-coated glass coverslips (BD bioscience, Bedford, MA, USA) and either treated for 10 min with 10 *μ*M of ionomycin (Calbiochem, EMD Biosciences, CA, USA) or left untreated (control). After the treatment, cells were washed twice with PBS, and fixed with 4% paraformaldehyde in PBS for 15 min at room temperature, followed by postfixation with methanol for 5 min at −20°C. The fixed cells were incubated with 0.3% Triton X-100 solution for 15 min at room temperature and the cells were blocked for 1 h with 2% FBS in PBS at room temperature. Subsequently, the cells were incubated with a rabbit anti-NGF polyclonal antibody (sc-548, Santa Cruz Biotechnology Inc.) (diluted 1 : 50) for 1 h at room temperature. After washing twice with PBS, the cells were incubated with a goat anti-rabbit fluorescein isothiocyanate-conjugated secondary antibody (81–6111, Zymed, Invitrogen Corporation) for 1 h at room temperature. The cells were washed and mounted in glycerol, and viewed under a laser scanning confocal microscope (Axiovert 200 M, Carl Zeiss Co. Ltd, Germany).

### Cell migration assay

Cell migration was determined by a micropore chamber assay. HSA/c cells (3.0 × 10^4^) were seeded onto the top chamber of a 24-well micropore polycarbonate membrane filter with 8-*μ*m pores (Becton Dickinson Labware, Lincoln Park, NJ, USA) in serum-free Ham's F-12/RPMI 1640 with 0, 1, 3, 10 *μ*g ml^−1^ of rabbit polyclonal anti-human *β*-NGF antibody (500-P85, Peprotech EC, London, UK) for neutralisation of NGF activity, with normal rabbit IgG (500-P00, Peprotech EC) for control, or with 0, 100, or 300 nM of K252a (Alomone Labs Ltd., Jerusalem, Israel), an inhibitor of kinase activity of TrkA. The bottom chamber was filled with Ham's F-12/RPMI 1640 containing 5% FBS as a chemoattractant. After 22 h of incubation in a 5% CO_2_ humidified incubator at 37°C, the membranes were fixed and stained by Diff-Quik reagent (International Reagents, Inc., Kobe, Japan), and the cells on the upper surface were carefully removed with a cotton swab. Cell migration was quantified by counting all migrated cells in each membrane.

### Transient transfection of NGF-small interfering RNA

siTrio (THF27A-345), a mixture of three targeted small interfering RNAs (siRNA), was purchased from B-Bridge International, Inc. (Sunnyvale, CA, USA). Sequences of the oligonucleotide targeted to NGF were 5′-ggacuaaacuucagcauucTT-3′ (sense), 5′-gaaugcugaaguuuaguccTT-3′ (antisense); 5′-gggcagacccgcaacauuaTT-3′ (sense), 5′-uaauguugcgggucugcccTT-3′ (antisense); and 5′-ccacagacaucaagggcaaTT-3′ (sense), 5′-uugcccuugaugucuguggTT-3′ (antisense). siTrio (200 nM) or nonspecific RNA for control was transfected into HSA/c using Oligofectamine reagent (Invitrogen Corporation) and Opti-MEM I medium (Invitrogen Corporation) according to the manufacturer's instructions. To confirm the efficiency of siRNA, we extracted protein and RNA after 48 h after transfection. We subcultured cells for migration assay at the same time. For enzyme-linked immunosorbent assay (ELISA), 24 h after transfection the medium was replaced with serum-free Ham's F-12/RPMI 1640 and maintained for another 48 h. Samples were then obtained.

## RESULTS

### Expression of NGF and its receptors in OESCC specimens

**Figure 1:**
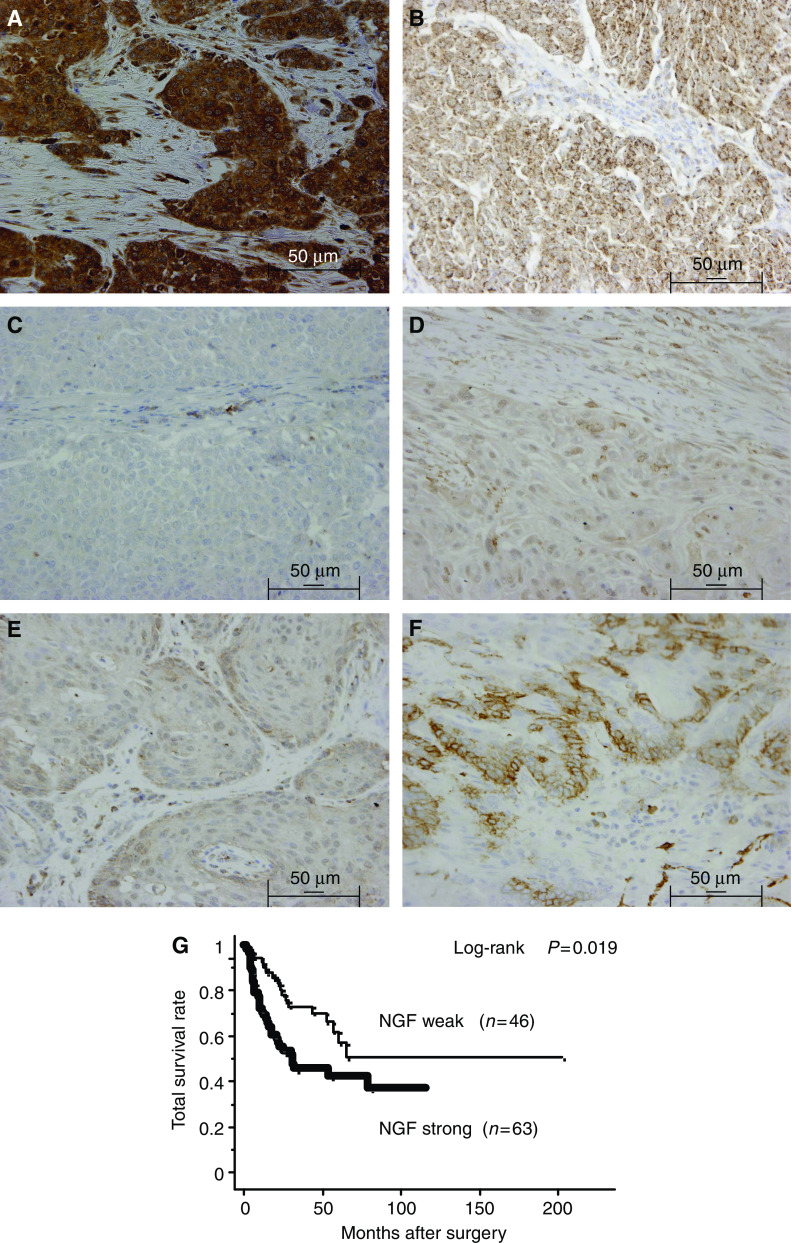
Typical immunohistochemical staining results of NGF, TrkA, and p75NTR for 109 OESCC patients. Each staining intensity was graded into two groups: (**A** and **D**) representative of strong and weak NGF expression, respectively; (**B** and **E**) representative of strong and weak TrkA expression, respectively; (**C** and **F**) representative of negative and positive expression of p75NTR, respectively. (**A**–**C**) are sections of poorly differentiated squamous cell carcinoma. (**D**–**F**) are sections of well-differentiated squamous cell carcinoma. Original magnification was × 400. Scale bar is 50 *μ*m. (**G**) Overall survival of patients classified according to the immunoreactivity of NGF. Overall survival analysis using the Kaplan–Meier method revealed that the survival of patients with tumours strongly expressing NGF was significantly poorer than that of patients with tumours weakly expressing NGF (log-rank *P*=0.019).

We first examined the expression of NGF in OESCC using immunohistochemical techniques. Consistent with the previous study (Zhu *et al*, 2000), NGF was expressed in the cytoplasm of OESCC cells and normal epithelial cells. Nerve growth factor staining intensity was graded into two groups; strong and weak expression (Figure 1A, D). We also examined the expression of TrkA (Figure 1B, E) and p75NTR (Figure 1C, F). Among the 109 OESCC specimens, 63 (57.8%) specimens had strong NGF expression, 76 (69.7%) specimens had strong TrkA expression, and 51 (46.8%) specimens had positive p75NTR expression. Correlations between NGF expression and various prognostic factors, such as pTNM pathological classification, histopathological grading, stage grouping, curability, and expression of TrkA and p75NTR were investigated (Table 1). Strong NGF expression was associated with positive lymph node metastasis (*P*=0.005), positive distant metastasis (*P*=0.017), poorer tumour differentiation (*P*=0.033), and higher TNM staging (*P*=0.025). Moreover, strong NGF expression was significantly associated with strong TrkA expression (*P*=0.032) and negative p75NTR expression (*P*=0.033). There was no significant association between NGF expression and other factors including age, gender, extent of the tumour, and tumour location. Calculation of survival by the Kaplan–Meier method revealed that strong NGF expression was significantly associated with poorer survival (*P*=0.019) (Figure 1C), but it was not an independent prognostic factor in multivariate analysis using Cox's regression model (data not shown). In this study, the only significant association found between either TrkA or p75NTR expression and clinicopathological factors investigated was the positive expression of p75NTR and negative lymph node metastasis (*P*=0.0009). TrkA and p75NTR were not shown to be prognostic factors (data not shown).

### Semiquantitative RT–PCR

**Figure 2:**
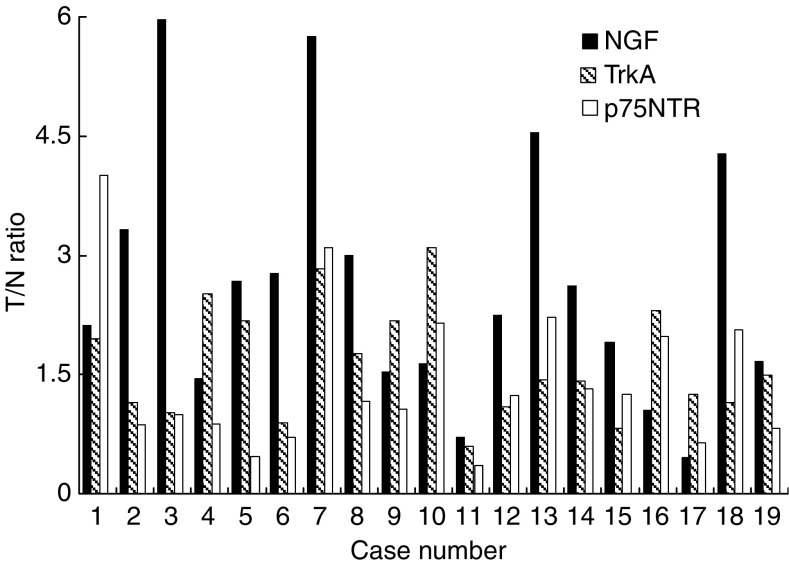
Semiquantitative RT–PCR. Ratio of NGF, TrkA, and p75NTR mRNA expression in the tumour portion (T) and corresponding normal epithelium (N) in 19 OESCC patients (T/N ratio) was examined. In 89% (17 of 19) of the patients, NGF mRNA levels were higher in the tumour portion and in 84% (16 of 19) TrkA mRNA levels were higher; 42% (eight of 19) of the pateints had lower p75NTR mRNA levels in the tumour portion.

To reveal the expression levels of NGF, TrkA, and p75NTR mRNA in OESCC, we analysed frozen tissue samples obtained from 19 patients with OESCC using semiquantitative RT–PCR, as described in Materials and Methods. Nerve growth factor mRNA expression was higher in the tumour portion than the normal epithelium portion in 89% (17 of 19) of the patients, and TrkA mRNA expression level was higher in the tumour portion in 84% (16 of 19) of the patients. The average tumour to normal epithelium expression ratio (T/N ratio) was 2.61±1.57 and 1.64±0.71, respectively, whereas 42% (8 of 19) had lower p75NTR mRNA level in the tumour portion, with average p75NTR T/N ratio of 1.43±0.94. The distribution of T/N ratio of NGF, TrkA, and p75NTR mRNA expression among the 19 patients is shown in Figure 2.

### Expression of NGF, TrkA, and p75NTR in OESCC cell lines

**Figure 3:**
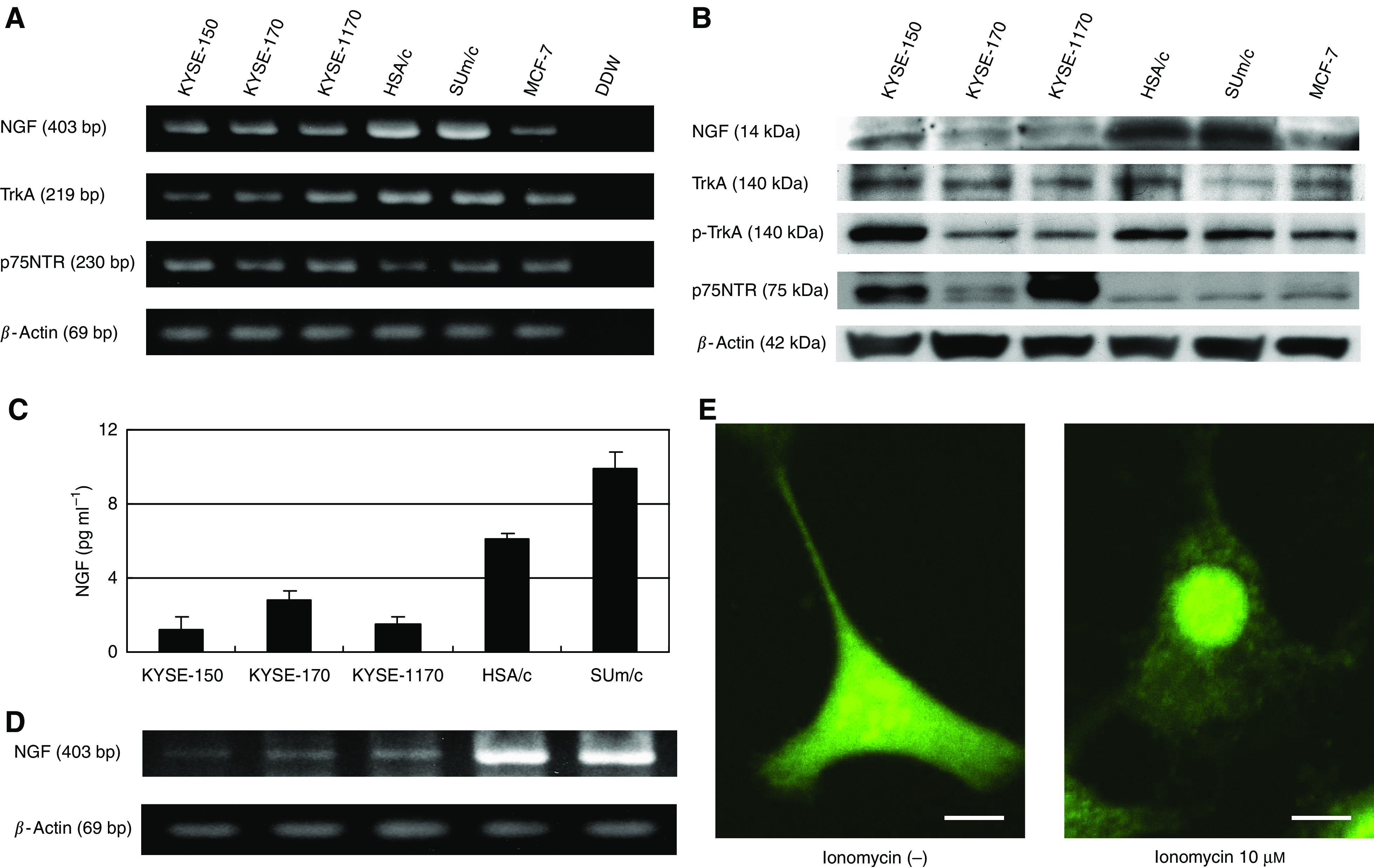
Expression of NGF and its receptors in five OESCC cell lines. (**A**) RT–PCR for NGF, TrkA, and p75NTR. NGF, TrkA, and p75NTR were all expressed in all five cell lines tested. Breast cancer cell line MCF-7 was used as a positive control. (**B**) Western blot analysis for NGF, TrkA, phospho-TrkA, and p75NTR. Although the expression levels of TrkA appeared to be similar among five cell lines, that of phosphorylated TrkA was clearly higher in KYSE150, HSA/c, and Sum/c, all of which expressed higher amount of NGF than the remaining two cell lines. (**C**) ELISA for NGF in OESCC cell lines (KYSE-150, KYSE-170, KYSE-1170, HSA/c, SUm/c) cultured in conditioned media. The secretion of NGF by all OESCC cell lines was clearly demonstrated. (**D**) RT–PCR of OESCC cell lines simultaneously performed with ELISA. RNA was extracted from the cells after conditioned media had been removed for ELISA, and used for RT–PCR. The amount of NGF secreted was positively related to the mRNA expression levels of NGF. HSA/c and SUm/c secreted relatively higher levels of NGF compared with the KYSE series. (**E**) Immunofluorescence staining of NGF. Confocal microscopy revealed the dramatically reduced NGF cytoplasmic concentration of HSA/c cells after treatment with 10 *μ*M of ionomycin (inducer of exocytosis). Scale bar is 10 *μ*m.

Next, we examined the expression of NGF, TrkA, and p75NTR in five OESCC cell lines (KYSE-150, KYSE-170, KYSE-1170, HSA/c, and SUm/c). The mRNA expression and protein expression of NGF, TrkA, and p75NTR were consistently detected by RT–PCR (Figure 3A) and Western blot (Figure 3B). Phosphorylated TrkA was also detected in all five OESCC cell lines on Western blot. Although the expression levels of TrkA appeared to be similar among five cell lines, the expression levels of phosphorylated TrkA was clearly higher in KYSE150, HSA/c, and Sum/c, all of which expressed higher amount of NGF than the remaining two cell lines. Specifically, the amount of NGF proteins detected in HSA/c and SUm/c was much higher than other three cell lines.

### NGF secretion from OESCC cell lines

The secretion of NGF by OESCC cell lines (KYSE-150, KYSE-170, KYSE-1170, HSA/c, SUm/c) was detected by ELISA, which clearly demonstrated that all OESCC cell lines secreted NGF (Figure 3C), and that the amount of NGF secreted was positively related to the mRNA expression levels of NGF (Figure 3D). HSA/c and SUm/c secreted relatively higher levels of NGF compared with the KYSE series. This finding was consistent with the results of Western blot analysis (Figure 3B).

To confirm that NGF is indeed secreted by viable OESCC cells, we used ionomycin, which is a known inducer of exocytosis (Kauffman *et al*, 1980), to reveal a release of NGF from OESCC cells. As shown in Figure 3E, after stimulation with ionomycin, cytoplasmic concentration of NGF was dramatically reduced, which strongly suggested that NGF was secreted by exocytosis.

### Effects of neutralising antibody against NGF and inhibitor of TrkA on motility of OESCC cell line

**Figure 4:**
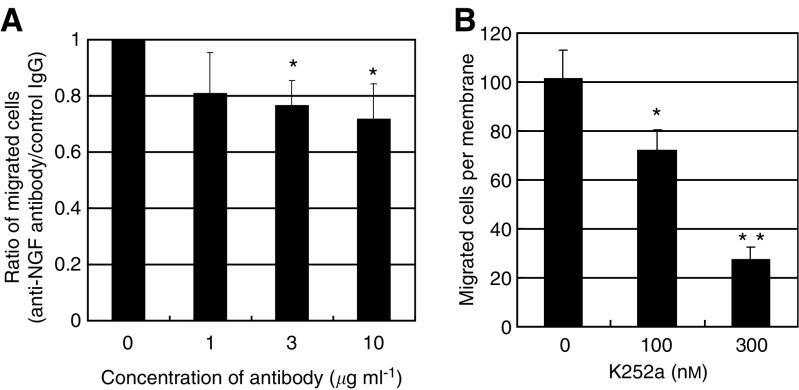
(**A**) Transwell migration assay of HSA/c with NGF neutralising antibody. The asterisk indicates significant difference (^*^P<0.05 the Tukey–Kramer test) *vs* 0 *μ*g ml^−1^. (**B**) Transwell migration assay of HSA/c with K252a (tyrosine kinase inhibitor for TrkA). K252a (100 nM) reduced cell migration significantly as compared with 0 nM. Moreover, 300 nM of K252a reduced cell migration significantly as compared with 0 and 100 nM. The asterisk indicates significant difference (^*^P<0.05, ^**^P<0.05, the Tukey–Kramer test).

To assess the role of NGF overexpression in OESCC cells, we used HSA/c, an OESCC cell line derived from lymph fluid in the thoracic duct and has a high potential for lymph node metastasis (Nakaji *et al*, 1999; Ito *et al*, 2006). Thus far, it has been revealed that HSA/c produced and secreted quite high amounts of NGF (Figure 3B-E). We blocked the NGF-TrkA pathway by using a neutralising antibody or K252a, a specific inhibitor of TrkA. Ten *μ*g ml^−1^ neutralising antibody of NGF decreased motility of HSA/c cells significantly to 70%, and 100 and 300 nM of K252a decreased migration of HSA/c cells to 70 and to 30%, respectively, as compared with control cells on migration assays, both reaching statistical significance (Figure 4A and B). We confirmed that neither neutralising antibody nor K252a affected the proliferation of OESCC cell lines (data not shown).

### Effects of transient transfection with NGF-siRNA on motility of OESCC cell line

**Figure 5:**
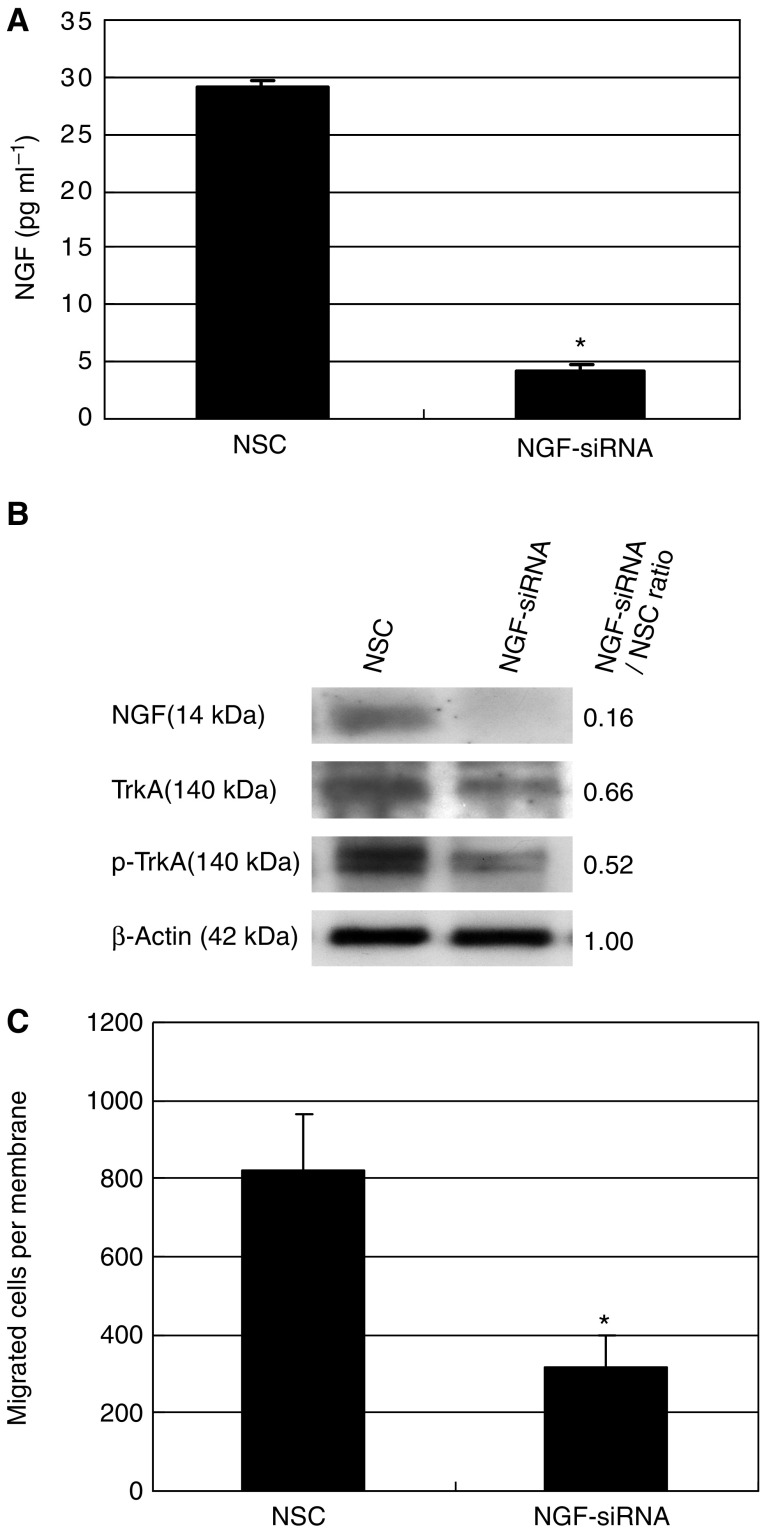
Transient transfection of HSA/c with NGF-siRNA. (**A**) NGF-siRNA significantly reduced NGF secretion from HSA/c by 85% in ELISA (^*^P<0.0001). Twenty-four hours after transient transfection of NGF-siRNA, medium was changed to serum-free medium. At 72 h after transfection, conditioned media were collected. NSC: nonspecific control RNA. (**B**) Western blot analysis for NGF, TrkA, and phospho-TrkA. After transfection with siRNA, the NGF expression decreased to the level nearly undetectable. Western blot also revealed reduction of phosphorylated TrkA. There was also reduced expression of TrkA after siRNA transfection, but its reduction rate is less than that of phospho-TrkA, as clearly shown by NGF-siRNA/NSC expression ratio. (**C**) Transwell migration assay. Nerve growth factor-siRNA significantly decreased cell migration (^*^P=0.0009).

To further confirm the role of NGF in cell motility, we used siRNA for transient transfection. The NGF expression level was efficiently reduced by 85% on ELISA (Figure 5A) and Western blot (Figure 5B). In fact, after transfection with siRNA, the NGF expression decreased to the level nearly undetectable by Western blot. Western blot also revealed reduction of phosphorylated TrkA (Figure 5B). There was also reduced expression of TrkA after siRNA transfection, but its reduction rate is less than that of phospho-TrkA, as clearly shown by NGF-siRNA/NSC expression ratio. Next, we examined the ability of NGF-siRNA treatment to affect cell migration. Downregulation of NGF dramatically reduced cell motility as compared with control cells; cell migration was decreased by 70% (Figure 5C). In the meantime, NGF downregulation did not affect cell proliferation (data not shown).

## DISCUSSION

In this study, we investigated the expression of NGF and its receptors in a large number of OESCC specimens and found that NGF was strongly expressed in 63 of 109 (57.8%) specimens. When the expression of NGF by OESCC was compared with clinicopathological data, it became apparent that strong NGF expression correlated with positive lymph node metastasis, positive distant metastasis, poorer tumour differentiation, higher TNM staging, and poorer survival. Furthermore, upregulation of NGF mRNA was also confirmed in 89% of OESCC patients.

There is only one study about the role of NGF in oesophageal cancer, showing that downregulation of NGF was associated with poorer tumour differentiation and advanced tumour stage (Zhu *et al*, 2000). However, among 41 patients of their study, nearly half of them had adenocarcinoma, thus number of OESCC patients is much lower than our study, which might lead to insufficient evaluation. On the contrary, as our results, most of previous reports regarding other cancers have shown the positive relationship between malignant potential of tumours and expression of NGF or its receptors (Geldof *et al*, 1998; Schneider *et al*, 2001; Zhu *et al*, 2001, 2002; Sakamoto *et al*, 2001a, 2001b; Kishibe *et al*, 2002; Davidson *et al*, 2003; Dolle *et al*, 2003).

The relation between two specific NGF receptors, TrkA and p75NTR, remains unclear. The expression of TrkA is considered a favourable prognostic factor in several types of cancer (Kramer *et al*, 1996; Combaret *et al*, 1997; Descamps *et al*, 2001a), whereas activation of TrkA by phosphorylation correlates with a poorer clinical outcome in serous ovarian carcinoma (Davidson *et al*, 2003). Results of the current study revealed that higher expression of NGF is correlated with increased production of phospho-TrkA in OESCC, and suppression of NGF expression resulted in decreased expression of phospho-TrkA *in vitro*. (Figures 3B and 5B). Moreover, immunohistochemical study of 109 OESCC clinical samples revealed that NGF strong expression was associated with strong TrkA expression and negative p75NTR expression (Table 1). We also performed semiquantitative RT–PCR for TrkA and p75NTR as well as NGF, which revealed that NGF and TrkA were expressed higher in the tumour portion than normal epithelium in 89 and 84% of patients, respectively, whereas p75NTR expression was lower in the tumour portion in 42% of patients (Figure 2). These results support our immunohistochemical study and imply that NGF-TrkA pathway is important for malignant potential of OESCC. Interestingly, progressive loss of p75NTR expression has been reported in more advanced lesions of several types of cancer (Pflug *et al*, 1992; Davidson *et al*, 2001, 2004). It may be possible that NGF stimulate TrkA rather than p75NTR in aggressive cancers with high malignant potential, as results of a study by Sakamoto *et al* (2001a) showed that patients with NGF+ and p75NTR− had poorer survival in breast cancer. Further study is needed to clarify whether NGF shifts its receptor to TrkA from p75NTR.

Next, we examined the role of NGF expression in OESCC cell lines. All five OESCC cell lines studied expressed NGF, TrkA, and p75NTR mRNA as well as protein. All of these OESCC cell lines showed TrkA phosphorylation on Western blot. Moreover, detectable levels of NGF were found in the conditioned media of the OESCC cell lines. The cellular motility was inhibited by NGF neutralising antibody, K252a (a TrkA inhibitor), and NGF-siRNA. The ability to inhibit cell motility was less with neutralising anti-NGF antibody than with K252a or NGF-siRNA, probably because it is difficult to neutralise NGF completely even with a specific antibody. Our results confirmed that the OESCC cell lines secrete biologically active NGF, which acts on TrkA in an autocrine manner to promote OESCC cell migration. These are compatible with our clinical findings from more than 100 cases of immunohistochemistry that overexpression of NGF is associated with lymph node metastasis and associated with poorer clinical outcome. To our knowledge, this is the first time to demonstrate NGF autocrine secretion in gastrointestinal cancer, although several previous studies have shown NGF autocrine secretion in other types of cancer (Weeraratna *et al*, 2000; Zhu *et al*, 2001, 2002; Dolle *et al*, 2003), as well as in noncancerous tissues (Torcia *et al*, 1996; Pincelli and Marconi, 2000).

One candidate molecule that promotes cell movement in the NGF pathway is Rho-guanine nucleotide exchange factor (Rho-GEF) Trio. It is known to be involved in the NGF pathway (Estrach *et al*, 2002), and activates RhoA with its GEF2 domain (Debant *et al*, 1996; Bateman and Van Vactor, 2001). Further investigation is necessary to unveil the involvement of Rho-GEF Trio in NGF pathway of OESCC cells.

Given the recent success of trastuzumab (Herceptin), imatinib mesylate (Gleevec), and gefitinib (Iressa) as chemotherapeutic agents, tyrosine kinase is definitely a promising target of molecular targeted medicine (Ross *et al*, 2004) for cancer therapy. Nerve growth factor–TrkA interactions could thus be a new therapeutic target. Nerve growth factor-siRNA might be one good option for OESCC treatment once tumour-specific siRNA delivering systems become available.

In summary, results of our immunohistochemical study of 109 OESCC patients clearly suggest that NGF is an unfavourable prognostic factor in OESCC. Furthermore, NGF–TrkA interaction promotes cellular motility in an autocrine manner, which in turn contributes to poor prognosis of NGF-secreting OESCC. However, it has also been shown that chemical agents that block NGF–TrkA interaction can inhibit cellular motility, leaving the possibility that these agents might be able to improve clinical prognosis of NGF-producing OESCC. These findings suggest that NGF is of potential interest not only as a prognostic factor, but also as a novel therapeutic target in OESCC.

## Figures and Tables

**Table 1:** Relations between NGF expression and clinicopathological characteristics

	**NGF strong (*n*=63)**		**NGF weak (*n*=46)**		***P*** **-value**
*Age (years)*					
<63	28	(25.7%)	27	(24.8%)	0.142
≥63	35	(32.1%)	19	(17.4%)	
					
*Gender*					
Male	52	(47.7%)	39	(35.8%)	0.755
Female	11	(10.1%)	7	(6.4%)	
					
*TNM*					
T1/T2	24	(22.0%)	21	(19.3%)	0.429
T3/T4	39	(35.8%)	25	(22.9%)	
N0	14	(12.8%)	22	(20.2%)	0.005
N1	49	(45.0%)	24	(22.0%)	
M0	46	(42.2%)	42	(38.5%)	0.017
M1^a^	17	(15.6%)	4	(3.7%)	
					
*TNM stage*					
I/IIA–B	22	(20.2%)	26	(23.9%)	0.025
III/IV	41	(37.6%)	20	(18.3%)	
					
*Histology*					
Well/moderately	42	(38.5%)	39	(35.8%)	0.033
Poorly	21	(19.3%)	7	(6.4%)	
					
*Location* b					
U	8	(7.3%)	9	(8.3%)	0.553
M	34	(31.2%)	21	(19.3%)	
L	21	(19.3%)	16	(14.7%)	
					
*TrkA expression*					
Weak	14	(12.8%)	19	(17.4%)	0.032
Strong	49	(45.0%)	27	(24.8%)	
					
*p75NTR expression*					
Negative	39	(35.8%)	19	(17.4%)	0.033
Positive	24	(22.0%)	27	(24.8%)	

a M1: All cases are with distant lymph nodes metastasis.

b U=cervical and upper thoracic oesophagus; M=middle thoracic oesophagus; L=lower thoracic and abdominal oesophagus.
